# Simulating the route of the Tang-Tibet Ancient Road for one branch of the Silk Road across the Qinghai-Tibet Plateau

**DOI:** 10.1371/journal.pone.0226970

**Published:** 2019-12-30

**Authors:** Zhuoma Lancuo, Guangliang Hou, Changjun Xu, Yuying Liu, Yan Zhu, Wen Wang, Yongkun Zhang

**Affiliations:** 1 Key Laboratory of Physical Geography and Environmental Process, College of Geography, Qinghai Normal University, Xining, Qinghai Province, China; 2 Key Laboratory of Geomantic Technology and Application of Qinghai Province, Provincial geomantic Center of Qinghai, Xining, Qinghai Province, China; 3 Department of computer technology and application, Qinghai University, Xining, Qinghai Province, China; 4 State Key Laboratories of Plateau Ecology and Agriculture, Qinghai University, Xining, Qinghai Province, China; University of North Carolina at Charlotte, UNITED STATES

## Abstract

As the only route formed in the inner Qinghai-Tibet plateau, the Tang-Tibet Ancient Road promoted the extension of the Overland Silk Roads to the inner Qinghai-Tibet plateau. Considering the Complex geographical and environmental factors of inner Qinghai-Tibet Plateau, we constructed a weighted trade route network based on geographical integration factors, and then adopted the principle of minimum cost and the shortest path on the network to simulate the ancient Tang-Tibet Ancient Road. We then compared the locations of known key points documented in the literature, and found a significant correspondence in the Qinghai section. However, there was a certain deviation between the key points recorded in Tibetan section and the simulated route; we found that the reason is the relative oxygen content (ROC) became a limited factor of the choice of the Tibetan section road. Moreover, we argue that the warm and humid climate and the human migration to the hinterland of the Qinghai-Tibet plateau were the fundamental driving forces for the formation of the Tang-Tibet Ancient Road.

## Introduction

**Fig 1:**
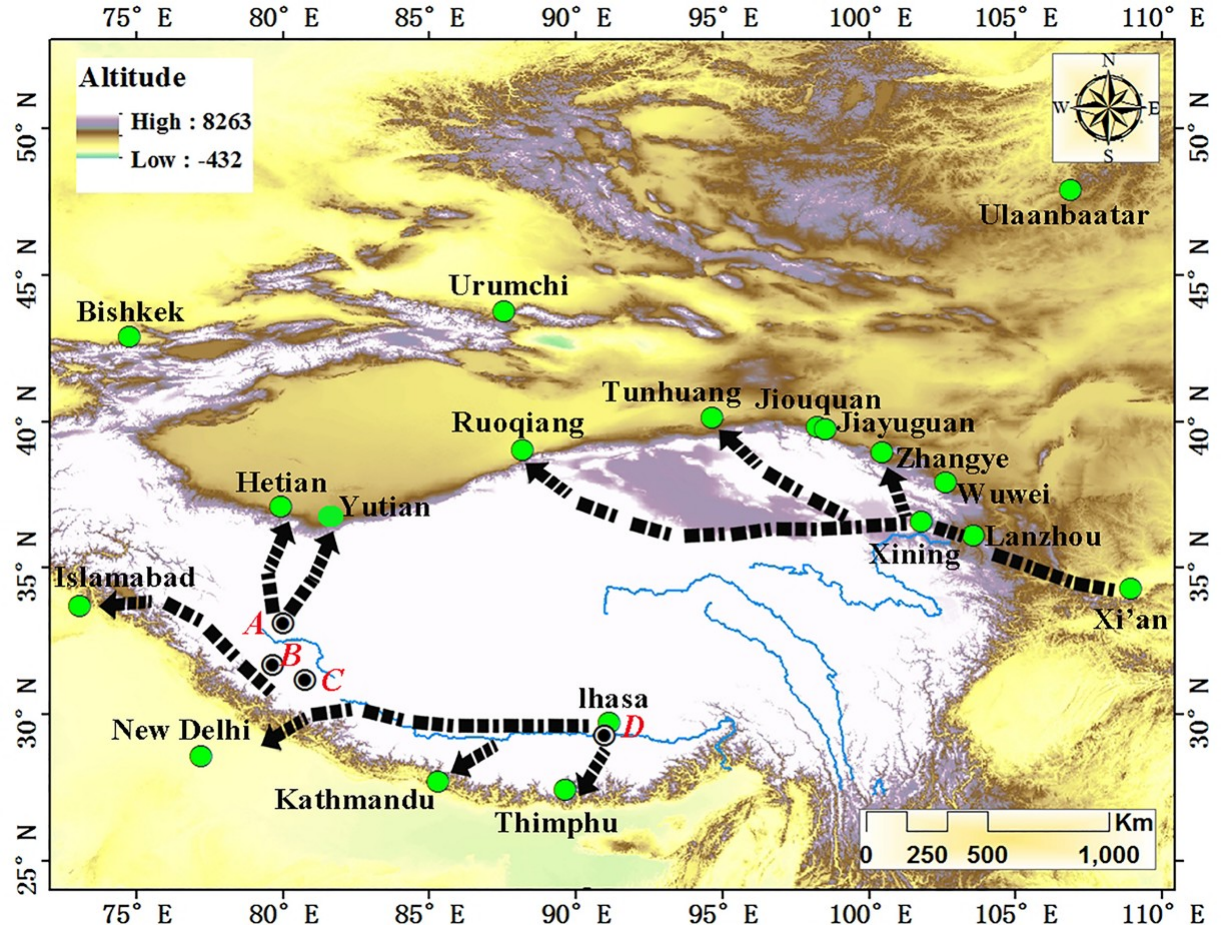
Trade routes on the edge of the Qinghai-Tibet Plateau connected with the Overland Silk Roads before the existence of the Tang-Tibet Ancient Road. Note: A, Evidence of Eurasian steppe style rock painting (Ritu Rock Art Sites in Ngari); B, Evidence of bronze mirrors with handles (Quta Cemetery in Ngari); C, Evidence of silk and tea (Gurgyam Cemetery in Ngari); D, Evidence of early metal (Qugong site in Lhasa).

The Belt and Road Initiative is designed to bean open, international network of cooperation and is becoming a multi-nation platform for exploring new mechanisms of international economic governance [1]. More specifically, the Overland Silk Roads (OSR) is a complex trade network that was formed by uniting large trading centers along with ancient transportation routes. Through this network, China has conducted extensive cultural and economic exchanges with Central, West, and South Asia [2–6]. The Qinghai-Tibet Plateau (QTP) is a key region connecting all of East, Central, West, and South Asia; owing to this geographical fact, this region has become the core of the Belt and Road Initiative [7–8]. Due to the harsh natural environment of the QTP, early trade routes existed around the margins of the QTP and historical and archaeological evidence (e.g., human activities, wheat and barley crops) from along these trade routes can be traced back to prehistoric times[9–14]. Using archeological evidence like bronze-handled mirrors (Archaeologists determined that such bronze-handled mirrors were introduced into the Tibetan Plateau in the early metal ages from West Asia and Central Asia. This is the evidence of communication between China and the west.), silk fabrics, tea leaves and Eurasian steppe-style rock painting, researchers have inferred that during the Han Dynasty (202 BC), people from the Southwest QTP reached the Silk Road through Xinjiang province (Yutian county) [15–18]. People from the Northeast QTP reached the Silk Roads via other roads, such as those going through Gansu province (Zhangye county and Tunhuang county) and Xinjiang province (Ruoqiang county) (Fig 1) [19–21]. These routes were all located on the geographical edge of the QTP. Up until the Tubo Dynasty (TBD) (618 AD-842 AD), these routes were important trade routes constituting OSR in the QTP [22–26].

Before the formation of Tang-Tibet Ancient Road (TTAR), both Qinghai and Tibet areas (today’s Tibet and Qinghai province) had their own separate routes to the OSR. However, there was no direct trade route in the inner plateau that could connect these two marginal areas. Given this, the TTAR is the only historically recorded Silk Road across the hinterland of the plateau. The road is approximately 2,240 km long, starting from Chang’an(today’s Xi'an province) and ending in Lhasa. Chen (1987) reported that the establishment of the TTAR connected the Xi'an-Qinghai and Tibet regions, which then linked OSR with the inner Plateau [27]. Hence, studies of the TTAR are crucial for a better understanding of the formative drivers and evolutionary processes of trade routes in the inner Plateau.

Currently, spatial data and geographic information systems (GIS) are widely applied to restore ancient trade routes [28–29]. For instance, Ma et al. (2017) used 30 m resolution Shuttle Radar Topography Mission (SRTM) data and adopted an optimal path analysis method to reconstruct the ancient OSR from Chang’an to Constantinople across the Eurasian continent [30]. Frachettiet al. (2017) used a flow accumulation model to reveal a high-resolution flow network that simulated how centuries of seasonal nomadic herding shaped discrete routes of connectivity across the OSR [31]. When combining these previous studies, these renderings relied primarily on terrain-based least cost to predict likely routes. This approach was effective in low altitudes OSR areas where economic networks and mobility between sites were consistent with human ease of travel [32–33]. However, this method may not be effective for the study of TTAR located in the inner QTP areas.

Previous studies on TTAR in the QTP areas relied primarily on historical literature and archaeological discoveries to determine node locations [34–38]. However, the path from node to node remains relatively unknown. Unfortunately, a better understanding of these paths is difficult, as the names of the nodes are quite different across historical records and modern literals due to linguistic changes. In particular, the lack of historical records has made it difficult to ascertain the route of the Tibetan section of the TTAR. Moreover, the complex and changeable environments (e.g., anoxia, low temperature, vast rivers, low vegetation primary production, and sparse population) of the plateau have increased the difficulty of obtaining high-resolution images of ancient Tibetan road paths. Therefore, it is difficult to obtain complete, high-resolution road maps of the TTAR.

In this study, we constructed a cost-response data set based on 90 m DEM (Digital Elevation Model) data (including slope, altitude, and relief data)[39], as well as data regarding river classification [40], accumulated temperature [41], vegetation [42], and population density [43]. We then established a weighted trade route network based node set. Based on this, a complete and high-resolution route of the TTAR was simulated using an optimal path analysis method. Finally, the accuracy and reliability of the simulation results were verified using historical records. This study sought to reveal the formative factors of ancient key trade routes inside the QTP, broaden our understanding of the relationship between the TTAR and the OSR, and strengthen human knowledge of extreme environments such as high altitudes and the resulting hypoxic conditions.

## Materials and methods

### Study area

The QTP has an average altitude of 4,400 m and covers an estimated area of 250 ×104 km2. The latitude is from 26°00′12″N to 39°46′50″N and its longitude is from 73°18′52″E to 104°46′59″E [44]. The altitude rises rapidly from approximately 2,000 m in the northeast to above 4,000 m in the hinterland of the plateau, forming a natural barrier for human entry. The primary landforms of the plateau are mountains, plains, and basins. The plateau includes the source of the Three-River areas—the Yangtze, Yellow, and the Lancang Rivers. The average temperatures of the coldest and hottest months are -10°C ~ -15°C and < 10°C, respectively. There are also dramatic diurnal and seasonal temperature variations [45–46]. The highest value of total solar radiation exceeds 8500 MJ/ (m^2^·a), and most parts of the plateau exceed 5000 MJ/ (m^2^·a) [47]. Along the TTAR in the QTP, vegetation zone communities mainly consist of subalpine forests, alpine meadows, alpine steppes, and alpine desert [48]. Low temperatures lead to insufficient soil nutrients, weak erosion resistance, slow plant growth, and low natural productivity [49]. In summary, our study area is characterized as an extreme natural environment that in ancient times presented a geographical barrier for human communication.

### Methods

**Fig 2:**
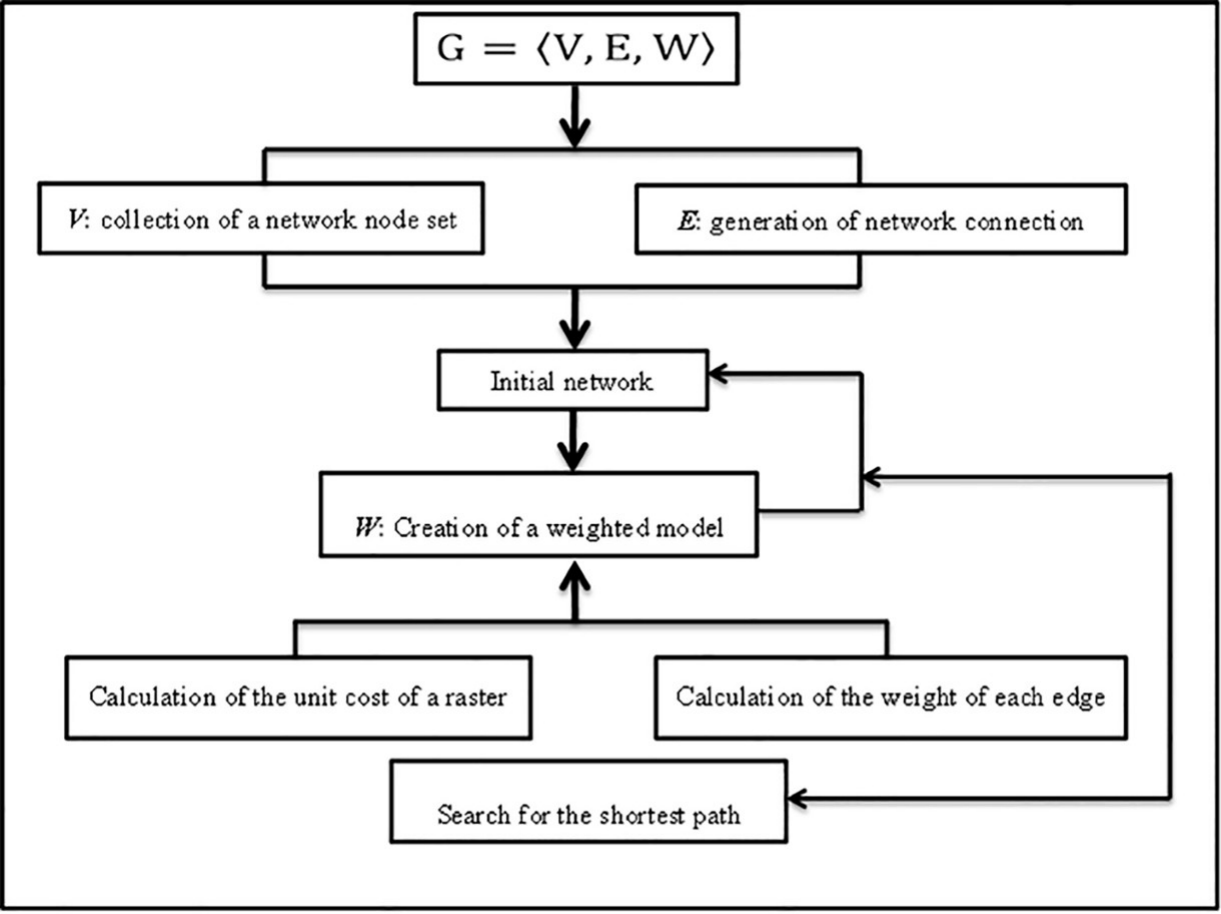
Flowchart for simulating the route of the Tang-Tibet Ancient Road.

In this study, we assumed that Xining and Lhasa were two separate trade centers, with the nodes along the route between the two centers functioning as courier stations. In accordance with these rules, we constructed a network Eq (1), which defined a node (courier stations) as the node of the network, and the route connecting the nodes as the side of the network. We then abstracted the problem into a path search algorithm on the weighted network. Based on the principle of minimum cost, the shortest path of the network was used to simulate the TTAR (starting node V_s_ [Xining] and the ending node V_e_ [Lhasa]) (Fig 2).

G=⟨V,E,W⟩ (1)

Where *V* is the node set, *E* is the network connection, and *W* is the weight. The method of this paper sought to solve the following four main questions: (1) collection of a network node set; (2) generation of network connection; (3) creation of a weighted model; (4) search for the shortest path.

#### Collection of a network node set

In this study, we assumed that nodes were courier stations along the aforementioned route. Therefore, we asserted the following types as nodes.

1. The site data showed direct evidence of the existence of ancient human activities. It is possible there was communication within and between the sites. The condition set was all sites data in the study area, which totaled to 512 [50] (“512” refers to the total number of archaeological sites found in the study area during the Tubo Dynasty (618 AD-842 AD) Available from: Atlas of Chinese Cultural Relics: Tibet Volume and Qinghai Volume.)
2. The criteria used for acceptable literature records regarding the TTAR selection used in this paper included the following: a chronology that spanned from ancient to modern literature documentation, literature types with official historical record, field investigation reports, and private travel notes. A total of 43 documented nodes were sorted; these node GPS data sets are shown in S1 Table. (Available from: documents [51–55])
3. Communication between inhabited settlements was the most frequent communication type in ancient times. Both modern and ancient settlements have a degree of historical continuity, but the scale of large and medium-sized modern settlements is quite different from those during ancient times. The number of modern small settlements may not be completely consistent with that of ancient times. After all, the increase of modern population is unsurpassed in any historical period, and the rapid increase of population requires human to develop more areas suitable for human survival, Because of this the number of small settlements must be far more than that of historical period. But now the small settlements must contain all the plateau settlements of the historical period, which also include the fixed central camps (Nomadic peoples settlements). Therefore, we used modern, township-level administrative settlements as node data. We totally collected 1,792 data points regarding the geographic location data of the township-level administrative centers(National Earth System Science Data Sharing Platform. The geographical location data of administrative centers at the township level 2012, Database: [Internet]. Available from: http://www.tianditu.gov.cn/[56])

#### Generation of network connection

This study was based on the following conditions: all nodes of a node set were connected, communication routes could be formed between each node, and that there was no intersection in the communication route. Accordingly, a spatial data model of the Triangulate Irregular Network (TIN) was used to create the communication route network [57]. The connected nodes formed a triangle in line with the Delaunay standard, i.e. the collection conformed to a series of connected—but non-overlapping—triangles, and the outer circle of those triangles did not contain any other points in the polygon. We required that the maximum TIN margin length of each node did not exceed the calculated value of 22 km. Of note, the sighting of the TBD courier stations was usually 22 km (Note: During the Tang Dynasty and the Tubo Dynasty, the communication between the central government and the local governments was completed through special postal caravanserais system. The post caravanserais established at that time had special mileage regulations, which roughly translate into the present mileage of 22 km. In other words, the government stipulated that the distance between the post stations was equivalent to 22 km [58]). This study combines the actual mileage of post stations in the Tang Dynasty and Tubo Dynasty when setting the maximum side length of the model. Therefore, we required that the maximum TIN margin length of each node did not exceed the calculated value of 22 km). The step adapts the 3D analyst tool ‘data management-TIN’ in ArcGIS10.5version (ESRI Corporation, Redlands, CA, USA).Because the connection route was on the ground plane, the field with altitude value (z) was defined as 0 when the node parameter was set and the final output was a spatial network that consisted of edges.

#### Creation of a weighted model

In this study, the creation of a weighted model sought to solve the following three main questions: (1) calculate the weight of each edge; (2) calculate the unit cost of a raster; (3) calculate the length of edge through raster.

**1. Calculation of the weight of each edge:** The weight of each edge was determined as follows: if: *p*_1_*p*_2_∈*V* and there are edges (existing routes) between *p*_1_ *and p*_2_, the edge is $e_{p_{1}p_{2}}=(p_{1},p_{2})\in E$, the cost function at coordinate (*x*,*y*) is *f*(*x*,*y*), and then the total cost of edges can be expressed as follows Eq (2):

$$W_{e_{p_{1}p_{2}}}=\int_{e_{p_{1}p_{2}}}f(x,y)ds$$ (2)

If $e_{p_{1}p_{2}}$ passes through *m* rasters, and the inside length of each raster is *L*^(*i*)^(*i* = 1,2,3,⋯*m*), then the unit cost of edges ($W_{e_{p_{1}p_{2}}}$) and the unit cost of raster(*S*(*i*)) and can be expressed as follows Eqs (3 and 4):

$$W_{e_{p_{1}p_{2}}}=\sum_{i=1}^{m}S(i)L^{\left(i\right)}$$ (3)

$$S(i)=\sum_{j=1}^{7}c_{j}∙f_{j}(i)$$ (4)

Where: *S*(*i*) is the cost estimate of unit raster *i*, *c*_*j*_ is the weight of the parameter *j* and *f*_*j*_(*i*) is the weight of the parameter *j* in raster *i*.

**Calculation of the unit cost of a raster:**The unit cost of a grid was calculated using formula 4 with a cost response data set (*W*) of a grid as an influencing factor. The parameters were calculated using Application of Analytical Hierarchy Process (AHP)[59].

**Creating the cost-response data set:** The cost-response data include: geographical environment factors composed of Slope (A), river (R), altitude (H), relief (U), and accumulated temperature (T), socio-economic factors composed of vegetation net primary production (NPP) (N) and population density (P). The function of the resulting cost-response data set is as expressed in Eq (5).

W=f(A,R,H,U,T,N,P) (5)

**Parameters in the calculation:** Human road choice is related to accumulated, long-term experience. The AHP was used to make quantitative experience judgments (Table 1). The more accurate square root method was used to calculate the standard layer single factor weight, which was also used to test consistency. The result of the consistency test was CR = 0.05, CI = 0.05, with a smaller CI value indicating a better consistency. The overall CI and CR were less than 0.1 (detailed description in S1 Text.).

**Table 1:** Weight calculation result.

Quasi-measurement layer	Weights	Solution layer	Weights	Scheme layer weights Of each factor
**Natural factor**	0.7	A	0.35	0.25
		R	0.27	0.18
		H	0.20	0.14
		U	0.16	0.11
		T	0.03	0.02
**economy**	0.3	N	0.6	0.18
		P	0.4	0.12

**Calculation of the length of the edge through raster:** If: the coordinates of *p*_1_ *and p*_2_ are (*x*_1_*y*_1_) *and* (*x*_2_*y*_2_), and *p*_1_ *and p*_2_ constitute an equation of a straight line, then: the intersection coordinates of $\bar{p_{1}p_{2}}$ and each raster boundary can be calculated as follows Eq (6): If: $\bar{p_{1}p_{2}}$ has *m* intersection points with the rasters, and the intersection points are (*p*^(1)^,*p*^(2)^,⋯*p*^(*m*)^), Then: the coordinate of the intersection point *i* (*p*^(*i*)^) satisfy Eq (6).

$$\frac{{y-y}_{1}}{x-x_{1}}=\frac{y_{2}-y_{1}}{x_{2}-x_{1}}$$ (6)

**Fig 3:**
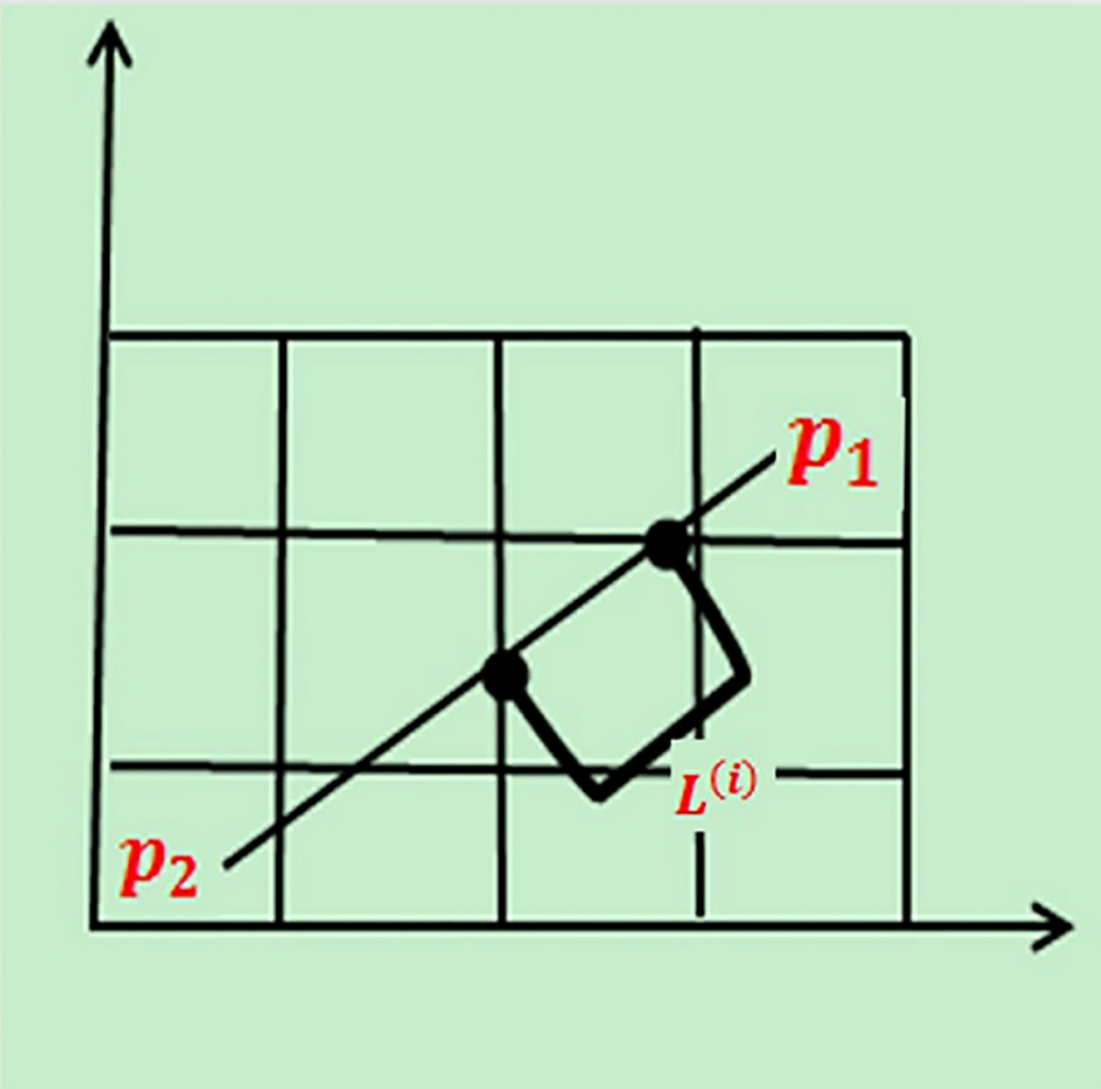
Calculation of the length (*L*^(*i*)^) of the edge through raster.

Eq (7) shows the calculation of the distance between *p*_1_ *and p*_2_ actually passing through the raster (*L*^(*i*)^ shown in Fig 3): the data set (*p*_1_*p*^(1)^,⋯*p*^(*m*)^*p*_2_) was sorted by x-coordinate size (*p*^(0)^,*p*^(*m*)^⋯*p*^(*m*+1)^) and the Euclidean distance (*D*) between two adjacent points was calculated.

$$L^{\left(i\right)}=D(p^{\left(i\right)},p^{\left(i+1\right)})$$ (7)

Where *D*(⋅,⋅)is the Euclidean distance and *i* is the intersection *i* after sorting.

#### Detailed description of data standardization

Table 2 provides the results of our data standardization grading value assignments. The cost-response data include: slope, river, altitude, relief, accumulated temperature, vegetation NPP, and population density. The detailed descriptions for data standardization for each are as follows:

1. **Slope:** Slope affects regional material flow and energy conversions, which are important factors impacting traffic lines [60]. First, this dataset considered the impact of the regional gradient on the road. Past studies have shown that humans cannot walk on a gradient of more than 70°; moreover, human activity has been shown to be mainly concentrated on terrain where the gradient is less than 40° [60]. The interval in the areas with a gradient of 0–40° was 5°, and the interval with gradient >40° was 10°. Values were assigned according to the following principle: The lower the slope, the lower the assigned value (Geospatial Data Cloud. Qinghai-Tibet Plateau DEM data product with 90m×90m spatial resolution. 2012, Database: [Internet]. Available from: http://www.gscloud.cn/)
2. **River:** Rivers are important supply routes. Within the study area there were a number of large rivers flowing from west to east, effectively blocking north-south traffic. The larger the river, the harder the crossing; comparatively, a small tributary river is more easily crossed and may also provide a source to replenish water supplies. The classification used was based on river order (1, 3, 4, and 5) and river buffer size (5 km, 7.5 km, and 10 km). The buffer zone design of rivers is that we think the ancient human walking passages mainly traveled on the river terraces, and did not sail against the water directly on the river. We divided the buffer zones into 5km, 7.5km and 10km for the convenience of water intake. Take level 1 rivers as an example. The terraces 5km away from the level 1 river are mainly V-shaped valleys with large slope, and water intake is not easy, therefore, the cost is relatively large. The terraces 7.5 km away from the river are relatively flat and easy to fetch water (In Level 1 Rivers, the flow is high; therefore an additional buffer value of 7.5km was added.). It is very difficult to get water 10km away from the river. The principle governing river assignment was as follows: the lower the river grade, the lower the assignment; similarly, the lower the river buffer from the river, the lower the assignment (National Earth System Science Data Sharing Platform. China 1:250000 first, third, fourth and fifth river grading data sets. 2002, Database: [Internet]. Available from: http://www.geodata.cn/)
3. **Altitude:** Medical research has shown that humans cannot survive at altitudes above 5,500 m for long periods of time; comparatively, when altitude is lower than 1,600 m, humans have no hypoxic response [61]. Past work has shown that when people enter the QTP for the first time, the incidence of acute high altitude diseases (AHAD) in the areas with altitudes ranging from 2,500–3,000 m is generally not more than 10%. When the altitude ranges from 3,000–4,000 m, incidence can exceed 30%, and from 4,000–5,000 m the incidence of AHAD is more than 50%. When the elevation is greater than 5,000 m, death can occur[62]. Therefore, elevation is a natural obstacle to human plateau traffic. In areas with altitude ≤ 4,000 m, the interval was 500 m, and in areas with altitude > 4,000 m, the interval was 250 m. These values were assigned according to the principle that the lower the altitude, the lower the value (Geospatial Data Cloud. Qinghai-Tibet Plateau DEM data product with 90m×90m spatial resolution. 2012, Database: [Internet]. Available from: http://www.gscloud.cn/).
4. **Relief:** Relief is an important index to quantitatively describe geomorphological form and can be used to divide the landscape into different geomorphologic types [63]. The level of fluctuation determines the role of the terrain in the choice of roads. This is because people will choose flatter plains for roads, to avoid as much as possible the high, undulating mountainous regions. Nine levels were used in this study to define relief: 0–30 m, 30–50 m, 50–70 m, 70–150 m, 150–300 m, 300–450 m, 450–600 m, 600–1000 m, 1000–2500 m, and >2500 m. The values were assigned according to the principle that the lower the relief, the lower the assigned value (Geospatial Data Cloud. Qinghai-Tibet Plateau DEM data product with 90m×90m spatial resolution. 2012, Database: [Internet]. Available from: http://www.gscloud.cn/).
5. **≥ 0°C accumulated temperature:** ≥ 0°C accumulated temperature is an important index to sub-divide the QTP into climatic zones [64]. Travelers entering the plateau had a poor adaptive response to the colder climate and preferentially chose areas with higher temperatures. Therefore, the accumulated temperature used was from high to low: 1200°C, 1100°C, 700°C, 500°C, 300°C, and 200°C for interval classification; the higher the accumulated temperature, the higher the value (Database Available from: ≥0°C accumulated temperature is obtained by the Chinese Academy of Agricultural Sciences, 1981–1990; Project sources: Database of the temperature and humidity data level in the background layer of China's ecological environment completed by the Agricultural Regional Planning Institute Database: [Internet]. Available from: http://www.caas.cn).
6. **Vegetation net primary production (NPP):** The NPP of vegetation refers to the total amount of dry, organic matter produced by plants in a unit of time and per unit area [65]. Given this, NPP can be used as an indicator of production for different vegetation types. The traditional economic model in this area is animal husbandry, so grassland has become an important seasonal productivity measure for QTP. For ancient travelers who had to carry a large amount of supplies, the supply of animal feed in long journeys was extremely important. Notably, animal feed could be provided by plants. Given the methodological complexity of accurately rendering vegetation changes in spatial distribution of grasslands, vegetation NPP data were most generalizable for reflecting the productivity level of vegetation cohorts under natural environment conditions in our model. The NPP can be used to effectively reflect the geographic distribution of QTP pasture resources associated with the productivity level in the animal husbandry economic system. Furthermore, the satellite image archive is limited to vegetation indices (NDVI) mapped from more than 30 years of data from MODND1D or AVHRR, so the longer-term averages are not necessarily better for modeling scalar changes in vegetation geography over the past 2000 years or more [66]. Thus, the NPP value we chose was not fixed per time and per unit area, but the fixed value with general significance calculated by combining vegetation types. Although not representative of every year over the past 2000 or more years, this vegetation NPP was reclassified according to the size associated with the productivity level of vegetation cohorts, for use as a generic modeling dataset. Our classification of vegetation types by NPP value was based on the calculation results of Chen et al. (2012), Zhou et al. (2004), and He et al. (2004) [67–69]. We predicted that the higher the NPP value, the higher the productivity level (Data Center of the Chinese Academy of Sciences by the Resource and Environmental Science. Spatial distribution data of 1 million vegetation types in China. 2012, Database: [Internet]. Available from: http://www.resdc.cn/data).
7. **Population Density:** Population density reflects the regional distribution of the population and is closely related to natural environment and level of socio-economic development [70]. Population density is not only an important indicator for gauging the level of human activities, but also an important factor in determining settlement location, which played a vital role in the development of ancient trade routes[71]. The areas with the highest population densities on the QTP are the Yellow River and the Huang Shui River Valley area in the eastern (Xining, 1000 people/km^2^) and the Lhasa River Valley area in the western (Lhasa, 1000 people/km^2^), which is consistent with the distribution pattern of population density in ancient times. The reason for this result is that the extreme environment limits the location of the settlement on the QTP, and the environment background in which human beings can settle down and live has relatively fixed[72]. Although the modern and ancient population densities cannot be exactly the same, the population density is a historical continuity, and the distribution of modern populations is a result of the past evolution of the regional population density. Therefore, modern population density data was used in this study. Our population density factor was classified based on various ranges of density rankings[73], with the higher population density giving a lower value. Briefly, more human traffic would be required in areas with higher population density, and thus these areas would be more possible to form roads. (Data cloud of Chinese academy of sciences. Population data of 1 km^2^ in China. 2000, Database: [Internet]. Available from: http://www.data.ac.cn/).

**Table 2:** Grading value assignments for all data standardization.

Geographical environment factors					Social-economic factors		
Slope/°	River	Altitude/m	Relief/m	≥0°Caccumulate temperature/°C	Population/km^2^	NPP(gC/m^2^.yr)	Value
0–5	5_10km	1600–2500	0–30	≥6500	>1000	Tropical rainforests, etc	1
5–10	5_5km	2500–3000	30–50	5300–6500	1000	Subtropical evergreen broadleaf forests (hard leaf), etc	2
10–15	4_10km	3000–3500	50–70	4200–5300	800	Subtropical coniferous forests, broadleaf mixed forests, temperate deciduous broadleaf forests, etc	3
15–20	4_5km	3500–4000	70–150	3500–4200	600	Subtropical evergreen broadleaf forests, etc	4
20–25	3_10km	4000–4250	150–300	2000–3500	500	Temperate coniferous forests, subtropical and tropical mountain coniferous forests, temperate deciduous small-leaf forests, etc	5
25–30	3_5km	4250–4500	300–450	1500–2000	400	Temperate deciduous shruband, etc	6
30–40	1_10km	4500–4750	450–600	1000–1500	300	Temperate grass and forb meadow, etc	7
40–50	1_7.5km	4750–5000	600–1000	800–1000	200	Cold-temperate and temperate mountain coniferous forests, alpine meadow, etc	8
50–70	1_5km	5000–5500	1000–2500	500–800	100	Dwarf trees desert, shrub desert, cushion dwarf semi-shrub alpine desert, alpine sparse vegetation, etc	9
>70	No river	5500>	>2500	<500	10	Alpine bog, desert, bare land, snow-capped land, saline soil, etc	10

(The lower the value, the lower the cost of human travel)

#### Search for the shortest path

**Fig 4:**
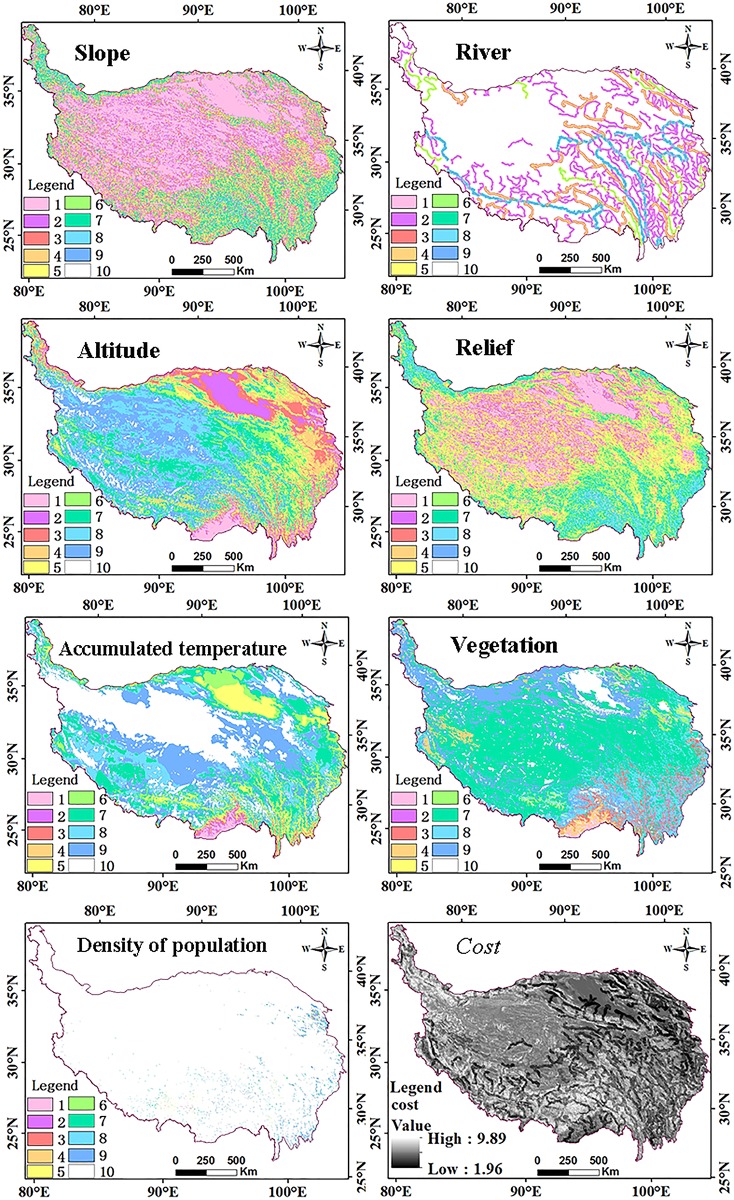
Raster data set after reclassification and cost weight.

**Fig 5:**
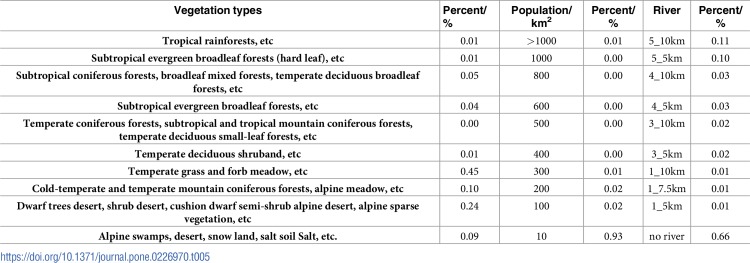
Weight result of each edge in the TIN.

The following process was implemented using the Arc GIS10.5 version (ESRI Corporation, Redlands, CA, USA). **Step 1: Conversion of cost data set to raster data.** Output raster dataset was based on the results shown in Table 2. **Step 2: Calculation of weight using raster calculator.** Based on the results in shown in Table 1 and Eq (8), the step 2 adapts the spatial analyst ‘map algebra-raster calculator’ in ArcGIS. A larger cost value indicates higher cost, and vice versa. (Fig 4) **Step 3: Calculation of weight of each edge in the TIN.** (Fig 5)

cost=0.25*A+0.18*R+0.14*H+0.11*U+0.02*T+0.18*N+0.12*P (8)

**Step 4: Search of the shortest route in the network.** The Dijkstra algorithm was used to calculate the shortest path from a source node to all other nodes. It is a classic algorithm that produces the shortest path in increasing order of path length [74]. Since the Dijkstra algorithm was used to search for the shortest path between two specified nodes in an existing network, the optimal route was calculated by setting Xining (*V*_s_) as the starting node and Lhasa (*V*_e_) as the target node. The **step 3** and **step 4** were executed in Python using ArcGIS’s native Python geoprocessing tools. The full programming codes necessary for executing the steps are available as a unified Python script in S2 Text.

**Step 5: Processing route.** We originally considered the network for the modeling (macro scale) to be a triangular network of straight lines, but the actual route (micro scale) was not straight lines. The path through each raster (micro-scale) cannot be a straight line, and these routes will also pass through the grid according to the actual cost. So we treated the routes that go through each grid. In order to produce a more accurate route, in this study, the model’s output result (macro-scale) was processed as close as possible to match the actual route (micro-scale). On the premise of transforming the raster into a matrix, the order of the matrix was equal to the number of rows of the raster multiplied by the number of columns of the raster. If it was a route, the value of the raster was 1, and the value of the element in the corresponding matrix was also 1; if it was not a route, the raster value was 0, and the value of the element in the corresponding matrix was also 0. Under this premise, a matrix was constructed, starting from the first route (*V*_1_) to the end of (*V*_n)_ (n = 52) Eq (9). The route formed by the cumulative sum was the simulation result (*R*).The step 5 was designed using the R Programming Language (Alcatel-Lucent Bell Labs, USA) script as shown in S2 Text.

$$R=\sum_{n=1}^{52}V_{n}$$ (9)

Where: **3070** is columns count of the raster, 1975 is rows count of the raster, and ***n*** is the route count.

## Results

### Simulation results of the Tang-Tibet Ancient Road

We used the comprehensive cost data as a weight to calculate the shortest path. Based on final results from our online search, the shortest path from Xining (starting node) to Lhasa (ending node) was calculated with a total weight of 50 nodes. Data from the collecting node set included 29 settlement nodes, 15 document nodes, 7 site points, and a total weight of 5391 (Table 3). The route was the one with the smallest sum of weights and the shortest distance connecting Xining to Lhasa with a total length of 1356 km. The maximum and minimum distances between these 50 nodes were 108 and 1 km, respectively, with an average distance of 24.2 km between these 50 nodes. We confirmed that 14% of these 50 nodes had a distance between 50 and 79 km, while 41% had a distance between 20 and 50 km, the remaining 45% had a distance between 0 and 20 km.

**Table 3:** Output simulation result.

Area I	Area II	Area Ⅲ	Node	line distance/km	cost
**Qinghai**	Xining City	Xining City	Xining	0	0
			*Qingtang Sites*	3	5
			Dabaozi Town	12	25
			Xiaozhai Village	0	0
		Huangzhong County	Duoba Town	10	23
			Duoba Village	1	24
			Zhenhai Fortress	2	4
			Gonghe Town	11	31
		Huagyuan County	Heping Village	13	40
			Xiaogaoling	1	4
			Yaoshui Village	7	26
			Kesuer Village	7	10
			Riyue Village	3	13
	Hainan Prefecture	Gonghe County	Menggu Village	17	69
			Daotanghe Town	4	14
			Shazhuyu Village	35	132
			Xiakaligang Village	23	68
			Tiegai Town	31	102
		Xinghai County	Heka Town	23	79
			Daheba	27	98
			Wenquan Village	12	43
	Guoluo Prefecture	Maduo County	Kuhaitan	59	240
			Huashixia Town	40	170
			Huanghe Town	79	273
			Yeniu Gou	29	91
	Yushu Prefecture	Chengduo County	Qingshuihe Town	108	432
			Gaduo Town	43	195
			Anchong Town	24	110
		Yushu County	*Kalayanchang site*	27	110
		Zaduo County	Ziyeyunsongduo	64	25
			Zaduo County	51	221
			Aduo Town	23	246
**Tibet**	Naqu Area	Baqing County	Gongri Town	75	107
			Maru Town	70	333
			Baqing Town	31	288
		Naqu County	Benta Town	23	124
			Xiaqu Town	50	85
			Daqing Town	10	203
			Kuoma Town	31	33
			*Kangxing site*	38	142
			Luoma Town	32	164
	Lhasa City		Xiangmao Town	39	123
			Gulu Town	22	170
		Dangxiong County	Wumatang Town	37	68
		Linzhou County	*Langnagang site*	29	144
			Pangduo Town	17	138
			*Qiwajin site*	24	69
			Songchang Town	2	107
			*Nalinzha site*	12	45
			*Kongbuqi site*	1	9
			Duodi Town	18	90
		Lhasa City	Lhasa	6	23
**Total**	6	14	52	1356	5388

(Data from the collecting node set are indicated as follows: italicized indicates sites, underlined indicates literature record, black indicates settlements, and areas I, II, and Ⅲ are according to today’s provinces)

### Simulation results of the comprehensive cost

The calculation results of the weights showed that slope (25%) was the most important factor in determining the route of the South Asian corridor across the QTP. Other important factors in determining the corridor included: river (18%), NPP factors (18%), altitude (14%), population density (12%), relief (11%), and accumulated temperature (2%). The highest and lowest slopes through which the simulated route passed were 32.34° and 0.21°, respectively. The highest relief through which the simulated route passed was 551 m, with a minimum of 6 m; The maximum and minimum altitudes through which the simulated route passed were 5,400 m and 2,285 m, The highest and lowest accumulated temperatures through which the simulated route passed were 23,810°C and 2,096°C, respectively(Table 4).

**Table 4:** Statistical results of simulated route comprehensive cost value.

**Area Ⅱ**	**Slope/°**	**Altitude/m**	**Relief/m**	**≥0°Caccumulate temperature/°C**	**Cost**
**Xining City**	[0.30, 11.87],Avg.4.71	[2285, 2395],Avg.2348	[19, 120],Avg.54	[22738, 23810],Avg.23248	[3.24, 3.78],Avg.3.42
**Huangzhong County**	[6.12, 17.73],Avg.10.68	[2626, 2836],Avg.2914	[127, 284],Avg.208	[15584, 18978],Avg.16725	[3.99, 5.66],Avg.4.79
**Huagyuan county**	[0.76, 9.85],Avg.4.97	[3005, 3968],Avg.3428	[18, 232],Avg.122	[6996, 16288],Avg.11612	[5.00, 6.55],Avg.5.60
**Gonghe county**	[0.58, 6.51],Avg.2.63	[2874, 3562],Avg.3100	[12, 99],Avg.43	[12193, 20229],Avg.17442	[3.68, 5.90],Avg.4.79
**Xinghai county**	[0.62, 15.30],Avg.6.82	[3585, 4539],Avg.3835	[22, 266],Avg.167	[3661, 13142],Avg.8383	[3.56, 6.96],Avg.4.94
**Maduo county**	[0.21, 13.00],Avg.4.37	[4132, 4600],Avg.4342	[6, 249],Avg.90	[3753, 5551],Avg.4795	[4.02, 6.60],Avg.5.37
**Chengduo county**	[0.30, 28.43],Avg.10.28	[3940, 4864],Avg.4529	[15, 551],Avg.205	[2096, 11579],Avg.5480	[4.42, 7.69],Avg.5.81
**Yushu county**	[12.67, 15.65],Avg.14.40	[4435, 4815],Avg.4583	[219, 284],Avg.247	[4144, 7263],Avg.6213	[5.14, 6.63],Avg.5.72
**Zaduo county**	[2.20, 32.34],Avg.18.68	[4075, 5273],Avg.4635	[85, 543],Avg.309	[3290, 8699],Avg.6161	[5.08, 7.85],Avg.6.52
**Baqing county**	[3.45, 28.13],Avg.15.58	[4394, 5400],Avg.4817	[117, 487],Avg.282	[4265, 9829],Avg.6413	[4.51, 7.24],Avg.5.88
**Naqu county**	[0.72, 23.19],Avg.5.46	[4265, 5119],Avg.4626	[17, 322],Avg.123	[5665, 10225],Avg.8306	[4.10, 7.36],Avg.5.66
**Dangxiong county**	[1.05, 27.26],Avg.8.95	[4063, 5096],Avg. 4668	[30, 535],Avg.234	[5200, 13278],Avg.9010	[4.34, 6.60],Avg.5.70
**Linzhou county**	[0.96, 30.34],Avg.9.49	[3664, 4570],Avg.3995	[19, 386],Avg.167	[15690, 23067],Avg.20496	[4.74, 7.54],Avg.6.04

(The data acquired for this table were obtained by extracting the value to the point for every 10 km).

As shown in Table 5, 11% of the simulated route crossed 10 km buffer of grade 5 rivers, and 10% crossed 5 km buffer. This is in line with the convenience of travelling on the plateau, mainly relying on small rivers. With population density as a route choice determinant, 93% of the simulated route passed through an area with a population density of 10 people/km^2^. Population density is closely related to route choice. Temperate grass and meadows were the main vegetation determinants and accounted for more than 45% of total vegetation through which the simulated route passed. The vegetation type meets the needs of animals to obtain feed, and can supply the feeding needs along the route.

**Table 5:** Statistical results of simulated route comprehensive cost value.

**Vegetation types**	**Percent/%**	**Population/km**^**2**^	**Percent/%**	**River**	**Percent/%**
**Tropical rainforests, etc**	0.01	>1000	0.01	5_10km	0.11
**Subtropical evergreen broadleaf forests (hard leaf), etc**	0.01	1000	0.00	5_5km	0.10
**Subtropical coniferous forests, broadleaf mixed forests, temperate deciduous broadleaf forests, etc**	0.05	800	0.00	4_10km	0.03
**Subtropical evergreen broadleaf forests, etc**	0.04	600	0.00	4_5km	0.03
**Temperate coniferous forests, subtropical and tropical mountain coniferous forests, temperate deciduous small-leaf forests, etc**	0.00	500	0.00	3_10km	0.02
**Temperate deciduous shruband, etc**	0.01	400	0.00	3_5km	0.02
**Temperate grass and forb meadow, etc**	0.45	300	0.01	1_10km	0.01
**Cold-temperate and temperate mountain coniferous forests, alpine meadow, etc**	0.10	200	0.02	1_7.5km	0.01
**Dwarf trees desert, shrub desert, cushion dwarf semi-shrub alpine desert, alpine sparse vegetation, etc**	0.24	100	0.02	1_5km	0.01
**Alpine swamps, desert, snow land, salt soil Salt, etc.**	0.09	10	0.93	no river	0.66

**Fig 6:**
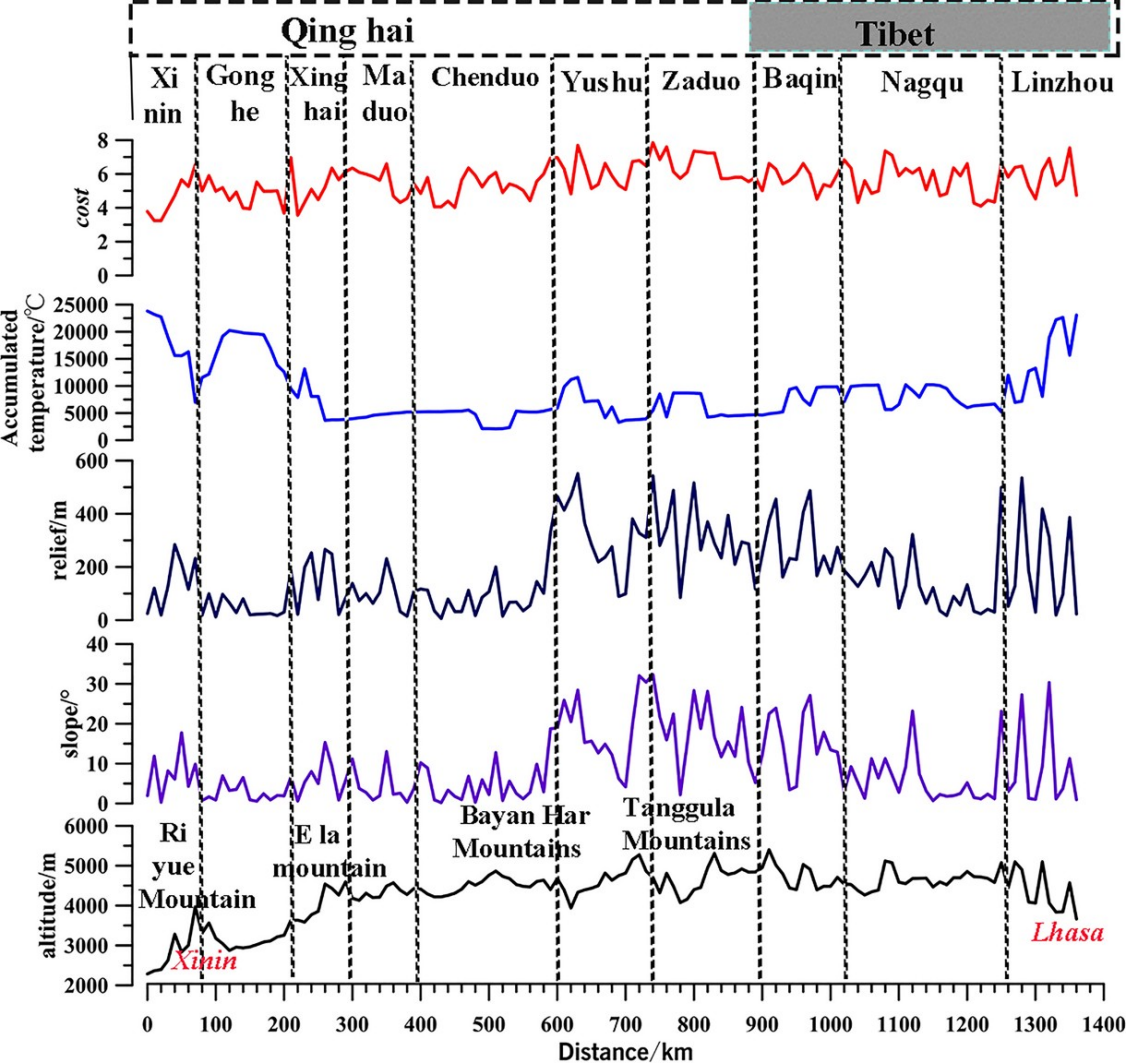
Relationship between district, distance, and comprehensive cost.

As shown in Table 6, with slope as a determining factor, a simulated route with a length of 180 km and passing over an approximate 20° slope accounted for 13% of the total route. The remaining 856 km of the simulated route passed over terrain with slope ≤ 10° and accounted for 63% of the total simulated route. As shown in Table 6 and Fig 6, the simulated route passed through relief areas between 100–200 m, which accounted for 320 km or 23% of the total simulated route. The simulated route passed through relief areas < 100 m, which accounted for a total length of 526 km or 39% of the total simulated route. A larger length (970 km) of the simulated route passed through areas with altitudes >4,000 m, which accounted for 71% of the total simulated route. This is very consistent with the topography of the study area. The slope and relief in over cross the high mountains is the highest. The section with the lowest terrain cost is concentrated in Qinghai province section. (Description of accumulated temperature of simulated route is explained in the discussion section: Comparison between simulated route and literature records**)**.The Pearson correlation coefficient was determined using the weight value of the simulated route and the route distance. The correlation coefficient R2 = 0.9782, which was significant (two-sided) at the 0.01 test level.

**Table 6:** Relationship between comprehensive cost, distance, and through the area in our simulated route.

**cost factor**	**Value**	**Distance/km**	**percent/%**	**Through the area**
**Slope/°**	>20	180	13	Bayan Har Mountains, Tanggula Mountains and the Naqu County entering Lhasa
	10_20	320	24	concentrated in the Zadu County crossing the Tanggula Mountains to Baqing County
	≤ 10	856	63	concentrated in the Gonghe and the Maduo County
**Altitude/m**	>5,000	90	7	concentrated in the Tanggula Mountains section
	4000 _5000	970	71	concentrated in Maduo, Chengduo, and Zaduo County
	<4000m	296	22	mainly concentrated in Qinghai Province
**relief/m**	400_551	120	9	They are concentrated in the Bayan Har Mountains, Tanggula Mountains and NaquCounty into Lhasa
	300_400	120	9	They are concentrated in the Bayan Har Mountains, Tanggula Mountains and Naqu into Lhasa
	200_300	270	20	concentrated in Maduo, Chengduo, Zaduo and Baqing County
	100_200	320	23	Concentrated in TibetProvince
	<100	526	39	Concentrated in Qinghai Province
**accumulated temperature/°C**	23810_10000	370	28	Concentrated in Xining, Gonghe County and Lhasa
	4000_10000	850	63	from Xinghai County to Lhasa, and along the line are distributed
	<4000	130	9	Concentrated in the Bayan Har Mountains and Tanggula Mountains.
**cost**	6_7	480	35	Concentrated in Zaduo, Yushu, Chengduo, lingzhou, Baqing, and NaquCounty
	4_5	810	60	from Gonghe, Maduo, Xinghai County
	<3	70	5	Concentrated in the Xining, Huangzhong, and Huangyuan County.

## Discussion

### Comparison between simulated route and literature records

**Fig 7:**
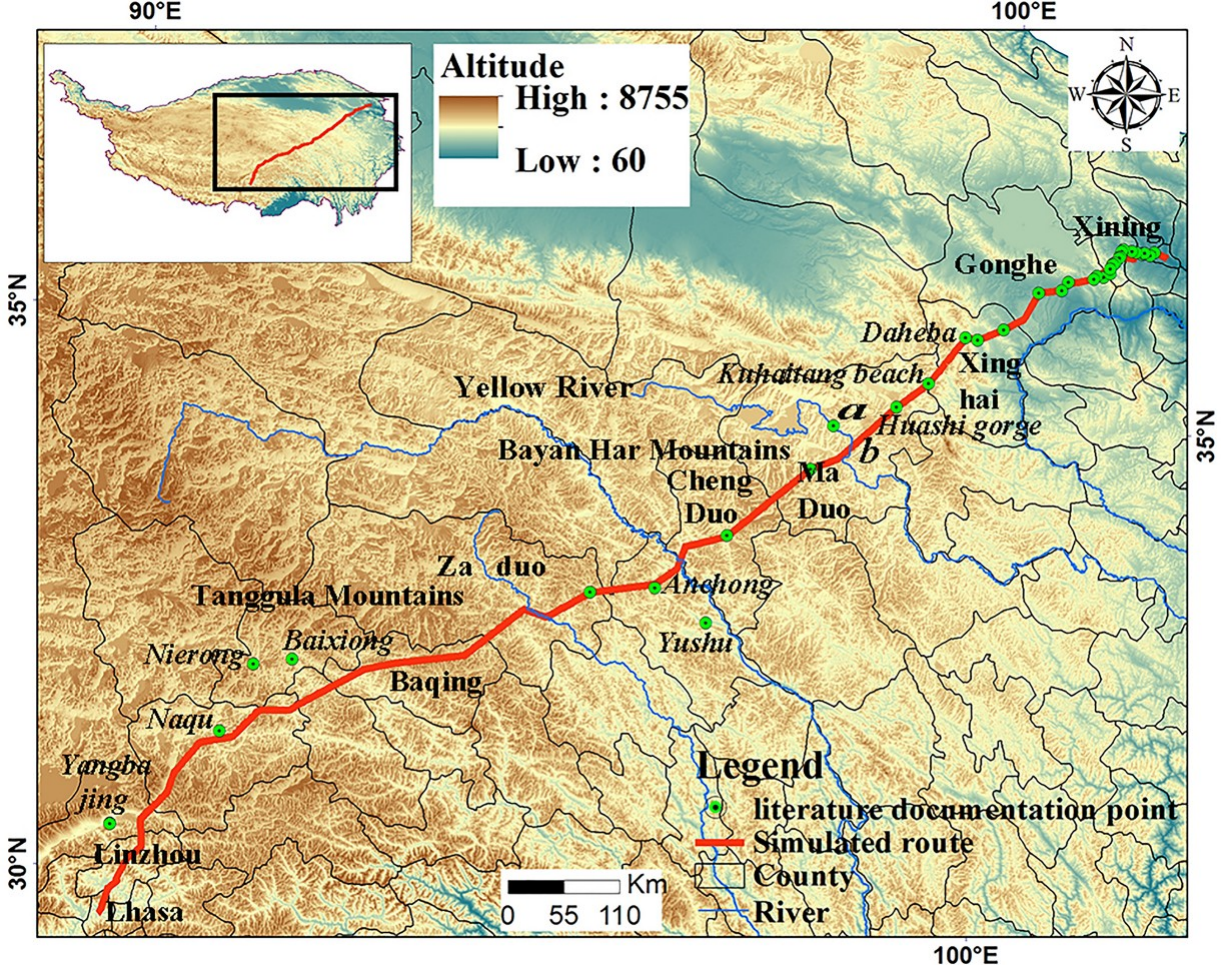
Comparison between simulated routes and key points in the literature.

According to the literature [53–55], the length of the TTAR is known to be 2,240 km. Table 7 shows the comparative results of the simulated TTAR distance with that found in the literature. These comparisons were composed of six sections of distance, including Xining-Xinghai County (X-X), Xinghai-MaduoCounty (X-M), Maduo-Chengduo County (M-C), Chenduo-YushuCounty (C-Y), Yushu-Zaduo County (Y-Z), and ZaduoCounty-Lhasa (Z-L). The distances of the first two sections (X-X and X-M) were almost identical to those from the literature. Moreover, in the first two sections, the key station nodes (Daheba, Kuhaitan, and Huashixia) that were documented in the literature also appeared in our simulated routes (Fig 7). The upper Yellow River lies in the M-C section. According to the literature, there were two ferries (Fig 7***A*** and 7***B***) that crossed the Yellow River. The simulated route crossed the Yellow River at ferry b, owing to the shorter distance and lower cost relative to ferry a. In the M-C section, the distance of our simulated route was 79 km less than that recorded in the literature. This may be due to the choice of different routes over the Bayan Har Mountains. Across the entire route, the simulated mountain crossing routes were always shorter than those obtained from the literature. This was because the simulated route followed the principle of shortest distance and lowest cost. This was also the reason why the distance of the simulated route crossing the Tanggula Mountains was much shorter than that recorded in the literature. The distance of the simulated route from ChengduotoYushu County (C-Y section) was 134 km less than that recorded in the literature. This was because the route documented in the literature passed through Yushu, which was an important economic and trade center in this region. However, the simulated route only passed through Anchong Township located in the north of Yushu, 120 km away from Yushu (show in Fig 7). Further, passing through Anchong Township is in line with the principle of the shortest distance and lowest cost.

**Table 7:** Comparison of distance between simulated routes and literature documentation.

**Comparative**	**Through the area**		**Literature** **Distance/km**	**Simulate** **Distance/km**	**Distinction** **Distance/km**
**Qinghai**	**X-X**	From Xining City to Huangzhong County	32	30	2	
		To Huangyuan County(Riyue Mountain)	78	70	8	
		To Gonghe County	49	50	-1	
		To Xinghai county	148	120	28	
	**X-M**	To Maduo county	233	200	33	
	**M-C**	Crossing Bayan Har Mountains to reach Chengduo county	249	170	79	
	**C-Y**	To Yushu county	164	30	134	
	**Y-Z**	To Zaduo county	233	140	93	
**Tibet**	**Z-L**	To overpass TanggulaMontains	450	190	260	
		To Naqu county	265	266	-1	
		To Lhasa	339	90	249	
**Total**		**Xining to Lhasa**	**2240**	**1356**	**884**	

In the Z-L section crossing over the Tanggula Mountains, the distance between the simulated and the literature routes were different. Comparing the key points between the route recorded in the literature and our simulated route, we found that the Qinghai section had 38 key nodes while the Tibetan section had five key nodes recorded in the literature. In the Qinghai section, 55.26% (21/38) of the key points documented in the literature appeared in our simulated routes. In the Tibetan section, the five nodes (Baixiong, Nierong, Naqu, Dangxiong, and Yangbajing) from the literature showed deviations from our simulated route(Fig 7).

When comparing the five variables in the region model where the five nodes were located with the region where the simulated routes were located, only cumulative temperature was significantly different in the Z-L section (Table 8). The accumulated temperature found in the literature route was 5723.69°C higher than that of the simulated route. The simulated route passed through 370 km of areas with accumulated temperatures between 23810–10000°C, which accounted for 28% of the total simulated route. The simulated route passed through 850 km of areas with accumulated temperatures between 4000–10000°C, which accounted for 63% of the total and went from Xinghai County to Lhasa. Previous studies have suggested that the relative oxygen concentration (ROC) in the near-ground air shows no obvious changes at different altitudes, but that atmospheric pressure and oxygen partial pressure significantly decrease with increasing altitude [75–77]. Further research has also shown that aside from altitude, surface vegetation coverage and weather conditions may also have an impact on ROC. These previous results indicated [78] that altitude, vegetation coverage, and 500 h Pa-T accounted for a total of 65.5% of the total variance in ROC based on principal component analysis. Moreover, the individual variance interpretation rate for atmospheric temperature (500 h Pa-T) variance, vegetation coverage variance, and altitude variance was 33.1%, 28.5%, and 3.9%, respectively. Owing to the same altitude of the simulated route and the literature route, there was no significant difference in absolute oxygen concentration. However, since the accumulated temperature increased, ROC also increased.

**Table 8:** Comparison of Z-L cost factors between simulated results and literature documentation.

**Z-L**	**cost factor**	**Literature**	**Simulate**	**Distinction**
	**Dem\m\mean**	4419.71	4419.69	0.02
	**Accumulated temperature\°C\mean**	12900.41	7176.72	5723.69
	**Slope\m\mean**	6.38	10.01	-3.63
	**Relief\m\mean**	123	201.6	-78.6
	**Cost\mean**	5.52	5.77	-0.25
	**Vegetation NPP**	_	_	_
	**Population density**	_	_	_

In Tibet area, the ability of humans to adapt to high altitude hypoxic conditions decreased with an average altitude of 4,419 m. Given this, it was reasonable to forego the route with shorter distance—but low oxygen concentration(simulation routes)—and choose the route with longer distance—but higher accumulated temperature and oxygen concentration(Baixiong to Nierong and Yangbajing to Lhasa). Since the simulated route focused primarily on distance and cost, it was 508 km shorter than the route recorded in the literature. As the route to Tibet was closely related to difference in human adaptations and needs, there may be multiple routes for crossing the Tanggula Mountains to Lhasa.Our simulation revealed one such route.

Based on our findings, the constraint factors on people's choice of roads in complex, natural environments are complex. These factors also demonstrated that the model used in our research needs to be modified in order to adapt to the simulation of ancient routes at high altitudes. The simulation of the Qinghai section showed that the model was applicable for altitudes ranging from 2,200 m to 4,400 m. In the areas with an altitude higher than 4,400 m, additional influential factors need to be considered.

### The formation and evolution of the Tang-Tibet Ancient Road

**Fig 8:**
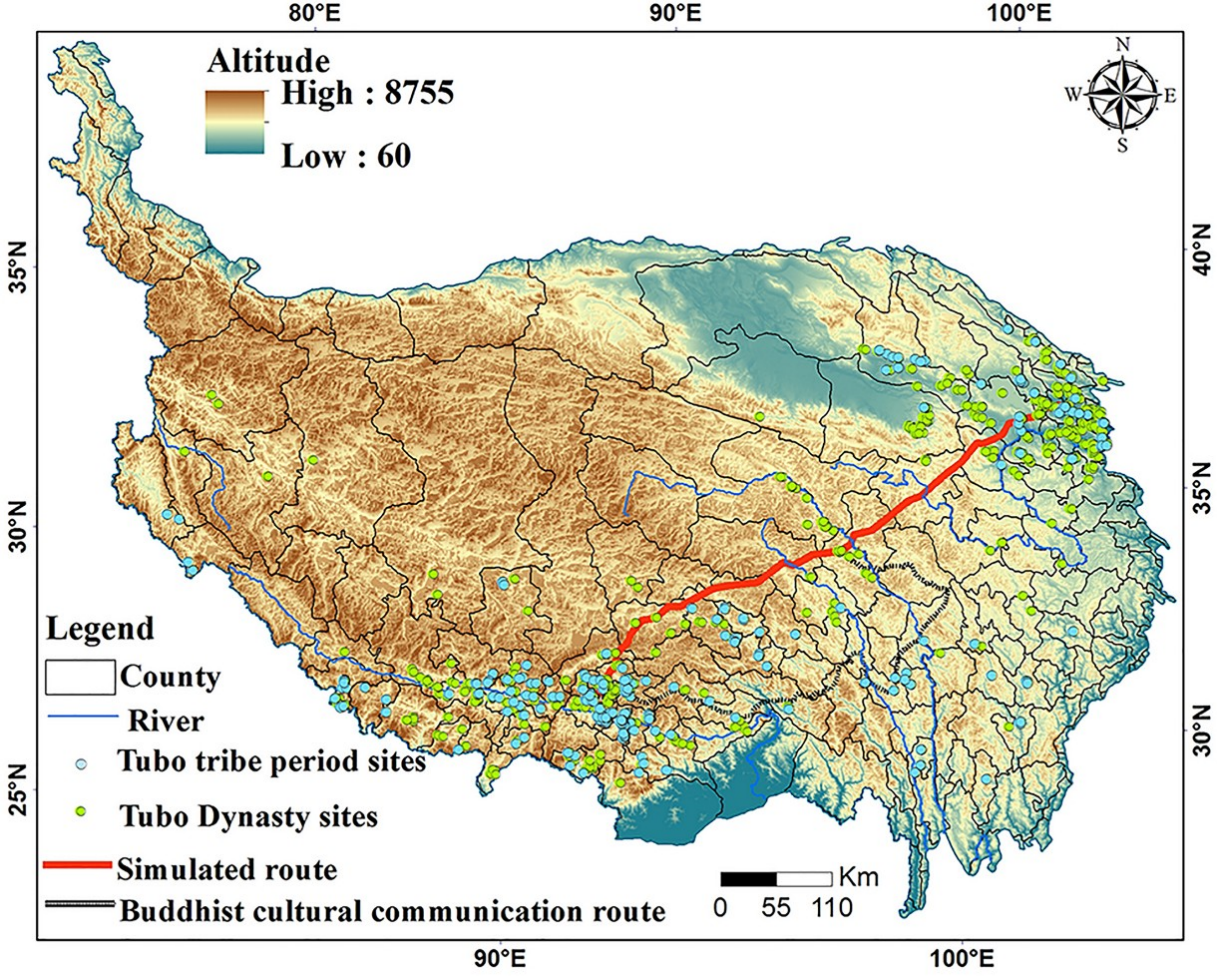
Distributions of simulated route, Buddhist cultural communication route, and sites.

A few studies have indicated that climatic changes during the Common Era were linked with the human history of China [79–80]. It is generally assumed that TTAR was formed during the Tang Dynasty (618 AD). This period also coincided with the rise of the Tubo Dynasty (618 AD-842 AD) (TBD). High-resolution climate reconstruction sequences have shown that the climatic characteristics of this period were warm and humid[81–82]. The response of the plateau vegetation to this climatic change was sensitive and rapid [83]. With an increase in temperature, the vegetation zones of the plateau expanded from the Southeast to the Northwest, from the edge to the hinterland, and from low to high altitudes. The increase in vegetation provided resources for nomadism and animal husbandry, which were the primary tribal economic activities in the heartland of the QTP[51–52]. Of the main vegetation types on the QTP, the range of alpine grasslands and meadows expanded during warm periods. This trend extended to high-altitude areas, which created the necessary conditions for the formation of trade routes. When this warm period ended, the vegetation belt moved wholly southward and the high-altitude routes were gradually abandoned. Given this, it is clear that during the cold period, the main route into Tibet was formed in the southern part of the QTP. Previous work has shown that the Buddhist cultural transmission route formed in the southern part of Tibet was also the main route to Tibet [55]. The formation time of this road coincides with the introduction of Buddhism into China, corresponding to the cold period of the Western Jin Dynasty (266AD-316AD)(Fig 8).

In addition, there was a correlation between climatic change, site spatial distribution, and site number. Corresponding to the cold period of the Tubo tribe (TBT, period before 618 AD) and the warm period of the TBD, the number of sites in the TBT period (261) was significantly less than the number of sites in the TBD period (512). The TBT sites were mainly distributed along the Southeast edge of the plateau, while the distribution of sites during the TBD expanded to the inner QTP (Fig 8). The reason for this trend in site expansion was that the warm and humid climate provided good climatic conditions for human activities. Therefore, the ancient road into Tibet gradually transitioned from the southeastern edge to the hinterland of the plateau. This trend was the result of plateau settlement and the expansion of human activities to the plateau hinterland given the warm climate of the medieval period.

## Conclusions

Here, we constructed a weighted trade route network, with data on slope, river, altitude, relief, accumulated temperature, vegetation NPP, and population density. We then used the principle of minimum cost and the shortest path on the network to simulate the Tang-Tibet Ancient Road. We found that slope, river, and vegetation NPP were the most important geographical determinants of the ancient road. Choosing a gentle slope, and routes along rivers and river valleys, and providing vegetation for feeding also corroborated that these three, major controlling factors must be present when traveling on the plateau. When comparing the simulated route with that obtained from the literature, the Qinghai section had a higher degree of fit in terms of both distance and direction; however, the Tibetan section deviated from this close fit. This deviation was due to ROC becoming a limiting factor when choosing the Tibetan section of the road. This finding further illustrated that avoiding hypoxia in high-altitude areas (4,400 m) was a fundamental factor for ancient human road choice. This also suggested that oxygen content in high-altitude areas needs to be fully considered in future models. Moreover, we argue that warm and humid climates as well as human settlement migration to the hinterland of the QTP were the fundamental driving forces for the formation of the Tang-Tibet Ancient Road. This evolution formed a stable settlement node in the center of the QTP. These nodes were like an island chain, running through the route connecting Xining to Lhasa and making the Tang-Tibet Ancient Road an important route for crossing the inner plateau.

## Supporting information

S1 Table GPS data set of the key points in the literature. (DOCX)

S1 Text Supplementary materials. (DOCX)

S2 Text Script programming codes. (R)
