# Supplementary material for: Simulating the route of the Tang-Tibet Ancient Road for one branch of the Silk Road across the Qinghai-Tibet Plateau
Source: PLoS One. 2019 Dec 30;14(12):e0226970. doi: 10.1371/journal.pone.0226970 (PMC6936881; doi:10.1371/journal.pone.0226970)
Supplement: S1 Table — (DOCX) [file pone.0226970.s001.docx]

**Supplementary materials 1**：GPS data set of key points in the literature(n.bArea I in the table: Refers to the prefecture-level city and the state-level administrative unit at the same level as the prefecture-level city. Area II: Refers to the county-level administrative units under the jurisdiction of prefecture-level cities and states.)

| Province | Area I | Area II | key point | X/º | Y/º |
| --- | --- | --- | --- | --- | --- |
| Qinghai | **Xining City** | **Huangzhong**  **County** | Dabaozi Town | 101.64 | 36.67 |
|  |  |  | XiaozhaiVillage | 101.6 | 36.64 |
|  |  |  | Duoba Town | 101.53 | 36.66 |
|  |  |  | Duoba Village | 101.52 | 36.66 |
|  |  |  | Zhenhai Fortress | 101.51 | 36.65 |
|  |  | **Huangyuan**  **County** | Zhama Long | 101.44 | 36.66 |
|  |  |  | Huangyuan Gorge | 101.39 | 36.67 |
|  |  |  | Dan Geer ancient city | 101.29 | 36.68 |
|  |  |  | Dongjia Village | 101.27 | 36.66 |
|  |  |  | Menggudao Village | 101.27 | 36.66 |
|  |  |  | Chahansu Village | 101.25 | 36.63 |
|  |  |  | Heping Village | 101.26 | 36.62 |
|  |  |  | Xiaogaoling | 101.25 | 36.61 |
|  |  |  | YaoshuiVilllage | 101.2 | 36.56 |
|  |  |  | Kesuer Village | 101.18 | 36.55 |
|  |  |  | Ruoyaotang Village | 101.15 | 36.5 |
|  |  |  | Shicheng site | 101.15 | 36.48 |
|  |  |  | Halakutu Village | 101.16 | 36.46 |
|  | **Hainan Prefecture** | **Gonghe County** | RiyueVillage | 101.08 | 36.42 |
|  |  |  | Menggu Village | 101 | 36.43 |
|  |  |  | DaotangheTown | 100.97 | 36.4 |
|  |  |  | GongheCounty | 100.62 | 36.28 |
|  |  |  | Suoerjia Village | 100.69 | 36.36 |
|  |  |  | ShazhuyuVillage | 100.36 | 36.25 |
|  |  |  | Qieji Reservoir | 99.86 | 36.08 |
|  |  | **Xinghai County** | Heka Town | 99.99 | 35.89 |
|  |  |  | Qinggenhe Village | 99.57 | 35.79 |
|  |  |  | Daheba | 99.71 | 35.78 |
|  |  |  | Wenquan Village | 99.51 | 35.5 |
|  | **GuoluoPrefecture** | **Maduo County** | Kuhaitan | 99.2 | 35.35 |
|  |  |  | Huashixia Town | 98.86 | 35.12 |
|  |  |  | Chali Town | 98.19 | 34.9 |
|  |  |  | Yeniu Gou | 97.98 | 34.49 |
|  | **YushuPrefecture** | **Chengduo County** | Qingshuihe Town | 97.14 | 33.81 |
|  |  | **Yushu County** | Jielong Town | 96.43 | 33.27 |
|  |  |  | Nianjiecuo | 96.24 | 33.09 |
|  |  | **Zaduo County** | ZiquGeishabiandi | 96.01 | 32.95 |
|  |  |  | Ziyeyunsongduo | 95.74 | 33.17 |
| Tibet Province | **NagQu Area** | **Naqu County** | Baixiong Town | 92.69 | 32.2 |
|  |  |  | Nierong County | 92.3 | 32.1 |
|  |  |  | Naqu City | 92.06 | 31.46 |
|  |  | **Dangxiong County** | Dangxiong County | 91.1 | 30.47 |
|  | **Lhasa City** | **Lhasa**  **City** | Yangbajing Town | 91.1 | 30.47 |

Note: The geographical location data of administrative centers at the townshiplevel\village level\countylevel and city. 2012, Database: [Internet]. Available from: http://www.tianditu.gov.cn/)
