# Supplementary material for: Simulating the route of the Tang-Tibet Ancient Road for one branch of the Silk Road across the Qinghai-Tibet Plateau
Source: PLoS One. 2019 Dec 30;14(12):e0226970. doi: 10.1371/journal.pone.0226970 (PMC6936881; doi:10.1371/journal.pone.0226970)
Supplement: S1 Text — (DOCX) [file pone.0226970.s002.docx]

1. **The weighting schema is vitally important in this paper. So, we give a more detailed description of the process of calculating:**

The detailed calculation process is as follows: Human road choice is related to accumulated, long-term experience. The Analytical Hierarchy Process (AHP) was used to make quantitative experience judgments. In this study, the cost-response data include: geographical environment factors composed of Slope (A), river (R), altitude (H), relief (U), and accumulated temperature (T), socio-economic factors composed of vegetation net primary production (NPP) (N) and population density (P). The more accurate square root method was used to calculate the standard layer single factor weight (Ep 1-2), which was also used to test consistency (Ep 3-4).

$$\boldsymbol{W}\boldsymbol{=}\sqrt[\boldsymbol{n}]{\left( \prod_{\boldsymbol{j}\boldsymbol{=}\boldsymbol{1}}^{\boldsymbol{n}} \boldsymbol{\alpha ij} \right)}\boldsymbol{,(}\boldsymbol{i}\boldsymbol{,}\boldsymbol{j}\boldsymbol{=}\boldsymbol{1}\boldsymbol{,}\boldsymbol{2}\boldsymbol{\cdots}\boldsymbol{,}\boldsymbol{n}\boldsymbol{)}$$

$${\boldsymbol{W}_{\boldsymbol{i}}\boldsymbol{=}\boldsymbol{w}}_{\boldsymbol{i}}\boldsymbol{/}\sum_{\boldsymbol{i}\boldsymbol{=}\boldsymbol{1}}^{\boldsymbol{n}} \boldsymbol{w}_{\boldsymbol{i}\boldsymbol{,}}\boldsymbol{(}\boldsymbol{i}\boldsymbol{=}\boldsymbol{1}\boldsymbol{,}\boldsymbol{2}\boldsymbol{,}\boldsymbol{\cdots}\boldsymbol{,}\boldsymbol{n}\boldsymbol{)}$$

**(Ep: 1-2)**

$$\mathbf{CI}\mathbf{=}\frac{{}_{\mathbf{max}}\boldsymbol{-}\boldsymbol{n}}{\boldsymbol{n}\boldsymbol{-}\boldsymbol{1}}\boldsymbol{=}\frac{\boldsymbol{-}\sum_{{}_{\mathbf{max}}} {}_{\boldsymbol{i}}}{\boldsymbol{n}\boldsymbol{-}\boldsymbol{1}}$$

$$\boldsymbol{CR}\frac{\boldsymbol{CI}}{\boldsymbol{RC}}$$

**(Ep: 3-4)**

(1): Construct judgment matrix according to expert scores. Table 1 is the judgment matrix and weights of geographical environment factors (G) and the socio-economic factors (S).

**Tab. 1 judgment matrix and weights (G-S)**

|  | G | S | W |
| --- | --- | --- | --- |
| G | **1** | **1\2** | **0.333** |
| S | **2** | **1** | **0.687** |

**λ= 2. CR= CI=0. (Weights Values rounding of 0.7 and 0.3)**

(2): Table 2 is the judgment matrix and weights of geographical environment factors.

**Tab. 2 Judgment matrix and weights of geographical environment factors**

|  | A | R | H | U | T | W |
| --- | --- | --- | --- | --- | --- | --- |
| A | **1** | **2** | **2** | **2** | **8** | **0.35** |
| R | **1\2** | **1** | **2** | **2** | **9** | **0.27** |
| H | **1\2** | **1\2** | **1** | **2** | **8** | **0.20** |
| U | **1\2** | **1\2** | **1\2** | **1** | **9** | **0.16** |
| T | **1\8** | **1\9** | **1\8** | **1\9** | **1** | **0.03** |

**λ= 5.207. CR=0.051, CI=0.046 (Values rounding of 0.05 and 0.05)**

(3): Table 3 is the judgment matrix and weights of socio-economic factors.

**Tab. 3 judgment matrix and weights (N-P)**

|  | N | P | W |
| --- | --- | --- | --- |
| N | **1** | **2** | **0.667** |
| P | **1\2** | **1** | **0.333** |

**λ= 2. CR= CI=0. (Weights Values rounding of 0.6 and 0.4)**

(4): According to the above results, the total weights of each factor are calculated. The total weights of each factor are the weight of the Solution layer multiplied by the weight of the Quasi-measurement layer to obtain the total weight of each factor. (Table 4).

**Table 4.Weight calculated result.**

| **Quasi-measurement layer** | **Weights** | **Solution layer** | **Weights** | **Scheme layer weights**  **Of each factor** |
| --- | --- | --- | --- | --- |
| **Natural factor** | 0.7 | A | 0.35 | 0.25 |
|  |  | R | 0.27 | 0.18 |
|  |  | H | 0.20 | 0.14 |
|  |  | U | 0.16 | 0.11 |
|  |  | T | 0.03 | 0.02 |
| **economy** | 0.3 | N | 0.6 | 0.18 |
|  |  | P | 0.4 | 0.12 |
